# Influence of seed concentration and storage time on the rheological, textural, and microscopic crystallization attributes of *Apis mellifera* honey

**DOI:** 10.1016/j.fochx.2025.102634

**Published:** 2025-06-04

**Authors:** Duygu Ozmen, Hüseyin Demircan, Rusen Metin Yildirim, Muhammad Waseem, Hakan Basdogan, Omer Said Toker, Crossby Osei Tutu

**Affiliations:** a Yildiz Technical University, Chemical and Metallurgical Engineering Faculty, Food Engineering Department, Istanbul 34210, Turkey; b Bursa Technical University, Faculty of Engineering and Natural Science, Department of Food Engineering, Bursa 16310, Turkey; c Department of Food Science & Technology, Faculty of Agriculture & Environment, Islamia University of Bahawalpur, 63100, Pakistan; d Erişler Food Company, Research and Development Center, Tekirdağ, Türkiye; e Department of Family and Consumer Sciences, University of Ghana, Legon, Accra, Ghana

**Keywords:** Honey crystallization, Seed concentration, Storage time, Rheology, Texture, Gastronomy

## Abstract

This study aimed to evaluate the impacts of seed honey supplementation at concentrations of 5, 10, and 15 % (*w*/w) and storage time (up to 12 days at 14 °C) on the rheological, textural, crystallization, and colour attributes of honey. The rheological properties, including the apparent viscosity, loss modulus, and storage modulus, were assessed using a rheometer. Textural properties, such as hardness and spreadability, were measured using a texture analyzer. The L* values were determined to assess the crystallization process, and microscopic imaging was used to observe the size and formation of the sugar crystals. Results showed that the apparent viscosity increased significantly (*p* < 0.05) from 13.1 Pa.s to 38.5 Pa.s (194 % ↑) as both the seed concentration and storage time increased from 0 %–15 % and 0–12 days, respectively. The highest *G"* value (2756 Pa) was observed on the 9th day for the 15 % seed concentration. The work of shear decreased with increasing seed concentrations but increased over storage time. L* values, an indicator of crystallization, showed a significant increase across concentrations and storage time. Microscopic examination of seed crystals anticipated zero crystals at 0–12th day in control; however, 6th and 12th days of storage at 10 % and 15 % addition of seeds increased size and magnitudes of crystals than 5 %. Conclusively, adding seed honey at levels up to 10 % and allowing 9 days of storage effectively promotes the shear-thinning behaviour desirable in spreadable honey products. Creamed honey offers improved texture and user convenience, enhancing product versatility without compromising flavour. These results provide a practical basis for optimizing processing conditions in commercial honey formulation to meet diverse consumer and market needs.

## Highlights

• Honey is a naturally occurring nutrient enriched food product.
• Creamed honey can enhance visual appearance of value-added foods.
• Viscosity, storage & loss modulus & colour improved with storage & crystallization.

## 1 Introduction

Honey, a sweet and viscous liquid, is a natural food produced by honeybees from plant nectars (Chen et al., 2009; Karasu et al., 2015). The dry matter of honey is mainly composed of two sugars, glucose and fructose, and essential constituents consisting of vitamins, minerals, enzymes, organic acids, volatile substances, proteins, health-promoting polyphenols, natural antioxidants, and essential amino acids (Al et al., 2009; Alvarez-Suarez et al., 2010; Scripca & Amariei, 2018; Yilmaz et al., 2014). The nutritional composition of honey undergoes alterations with the type of plant nectar, geographical origin, and climatic conditions (Dobre et al., 2012; Escuredo et al., 2013; Escuredo et al., 2014). Honey is well-recognized and preferred by consumers due to its health-promising nutritional features, antioxidant activities and pharmacological effects such as anticarcinogenic, antimicrobial and anti-inflammatory characteristics (Al-Waili, 2004; Tornuk et al., 2013; Yilmaz et al., 2014). Honey tends to crystallize during storage due to its high sugar content.

Crystallization is a complex natural phenomenon that occurs in honey during storage (Escuredo et al., 2014; Krishnan et al., 2021; Tappi et al., 2019) depending on the storage condition and honey composition. The crystallization in honey is primarily linked with the glucose in honey, owing to its lower solubility than that of fructose (Krishnan et al., 2021; Khan et al., 2025). Honey crystallization is direcly influenced by the fructose/glucose ratio, glucose/water ratio, pre-formed crystals, temperature of storage, and viscosity (Akonor et al., 2022; Dettori et al., 2018; Haider et al., 2025; Krishnan et al., 2021). The natural crystallization of honey results in a heterogeneous structure and coarse crystals, which adversely affects the edible quality, acceptability and processing, and marketing quality (Adil et al., 2025, Adil et al., 2025; Dettori et al., 2018; Subramanian et al., 2007; Waseem et al., 2024). Due to the higher humidity in the uncrystallized part, yeast growth is faster, which thereby reduces the shelf life of honey (Krishnan et al., 2021). The degree of crystallization affects the rheological and textural properties of honey and decreases consumer acceptance (Dettori et al., 2018).

Controlled crystallization of honey is used to produce a stable and homogeneous fine crystalline good quality product with improved sensorial, rheological and textural properties and shelf life (Chen et al., 2009; Dettori et al., 2018; Haider et al., 2025; Karasu et al., 2015; Suleman et al., 2025). Earlier literature has validated the use of the induced static crystallization technique involving the incorporation of 5–10 % (*w*/w) seed crystals (crystallized honey sample) in homogeniously mixed liquid honey at 14 °C (Chen et al., 2009; Dettori et al., 2018; Tappi et al., 2019). The induced static crystalization technique produces a uniform, spreadable, and creamy honey called creamed honey (Krishnan et al., 2021). Creamed honey is enriched with higher concentrations of fine-sized crystals and a smooth texture (Conforti et al., 2006). The crystallization of grape molasses with crystallized honey as a seed was achieved as a novel product to improve the spreadability of the molasses. In the study, it was reported that the storage time and seed concentration significantly affected the quality characteristics of the molasses (Ozmen et al., 2023). The results of the study reveal that it is possible to produce a more preferable product in terms of sensory properties without damaging the nutritional and health beneficial properties of similar products such as molasses and honey with an environmentally friendly process without any heat treatment or additional ingredients.

Considering gastronomy aspects, honey is used in many purposes such as dressings, sweeteners in desserts, beverages and sauces; marinades; flavour enhancing, health benefits and visual appeal. However, the high fluidity of honey limits some applications in gastronomy. It is possible to overcome some of these problems with the use of creamed honey. Crystallization can improve the spreadability of honey, and its thick and smooth texture makes it easy to spread on bread, toast, or pancakes without dripping. The creamy appearance of honey can enhance the visual appearance of dishes and presentations. The thick structure makes its taste less sweet than liquid honey, allowing for better control over sweetness in recipes. Crystallization can provide versatility in applications such as baking, toppings, fillings, and as an ingredient for dressings, sauces, and marinades. In conclusion, creamed honey can enhance culinary experiences by providing ease of use and versatility while maintaining the delightful flavour of honey.

Rheological and textural characteristics of creamed honey are considered significant attributes for consumers owing to its better sensorial acceptance, especially in terms of consistency and rheological properties, handling and processing. Cream honey has a similar spreadability character to butter or cream without dripping, making it more desirable for both the food industry and consumers (Akonor, Osei Tutu, Affrifah, Budu and Saalia, 2023a, Akonor, Osei Tutu, Affrifah, Budu and Saalia, 2023b; Asiedu et al., 2025; Chen et al., 2009; Karasu et al., 2015). This form of honey is appreciated by many consumers. However, meager amount of data are available on exploring the potential effect of seeding and storage on creamed honey. Therefore, the present study was planned to evaluate the effect of seeding at 5, 10, and 15 % (*w*/w) supplementation levels on the rheological, textural properties, and crystal formation of creamed honey after storage at 14 °C for 12 days. The results of the study can contribute to the industry and the literature on the production of cream honey with the desired quality.

## 2 Materials and methods

### 2.1 Sample preparation

A honey sample of *Apis mellifera* sp. was purchased from a local market in Istanbul, Türkiye, and crystallized honey was supplied from a local honey manufacturer (Altıparmak Food Industry and Trade Inc., Türkiye). From the crystallized honey, accurately measured 5, 10, and 15 % (w/w) were separately added in each honey sample and stirred manually for homogeneity. Honey sample without the addition of any crystallized honey (seed) was used as control. All samples were stored at 14 °C for 12 days. Analyses were performed every 3 days during the 12-day storage period.

### 2.2 Rheological analysis

The rheological behaviours of the honey samples were evaluated using a rheometer (Anton Paar, MCR-302, Austria) equipped with a parallel plate (PP-25) (diameter = 25 mm, gap = 1 mm) at 25 °C. To determine the flow behaviour, the shear rate was increased logarithmically from 0.01 to 100 s^−1^. The apparent viscosity at 50 s^−1^ was also recorded. In the frequency sweep test, the angular frequency was decreased logarithmically from 100 to 0.1 rad/s at a constant shear strain of 0.5 % within the linear viscoelastic range. To determine the viscoelastic behaviour, the elastic modulus (*G'*) and the viscous modulus (*G"*) were recorded.

### 2.3 Textural analysis

Texture analyzer (TA.HD Plus, Stable Micro Systems, United Kingdom) was used to evaluate the hardness and spreadability of the honey samples by obtaining the firmness and work of shear values. The stored honey samples were used immediately after they were removed from the refrigerator at 14 °C. As the device apparatus, a 45° cone Perspex and a 5 kg load cell were used. The trigger force, strain target, pretest speed, test speed, and posttest speed were adjusted as 5 g, 30 %, 1 mm/s, 3 mm/s, and 10 mm/s, respectively.

### 2.4 Colour analysis

In the colour analysis, the *L*^⁎^ (lightness / darkness) values of all honey samples were determined using a chroma meter (Konica Minolta, CR-400, Japan). A D65 illuminant was used as the light source. The calibration was performed using a white plate (L* 94.35, a* − 0.05, and b* 0.41).

### 2.5 Microscopic imaging

The particle sizes of the sugar crystals ranging between 0.01 and 20,000 μm were estimated using a particle size analyzer (Mastersizer 2000, Malvern, UK) equipped with a laser diffraction system. The sugar crystals' microscopic images at a magnification of 40× were taken using the light microscope (Olympus, BX53, Japan). A small droplet of honey was placed on a clean glass slide and covered with a cover slip. Observations were carried out under ambient conditions.

### 2.6 Statistical analysis

The results are expressed as means ± standard deviations (S.D.). Statistical analyses were performed in triplicates and tested using the JMP software version 13.2.0. One-way analysis of variance (ANOVA) was used to analyze the data, and differences among the mean values were evaluated using Tukey's honestly significant difference (HSD) test at *p* < 0.05 significance level.

## 3 Results and discussion

### 3.1 Rheological properties

**Fig. 1:**
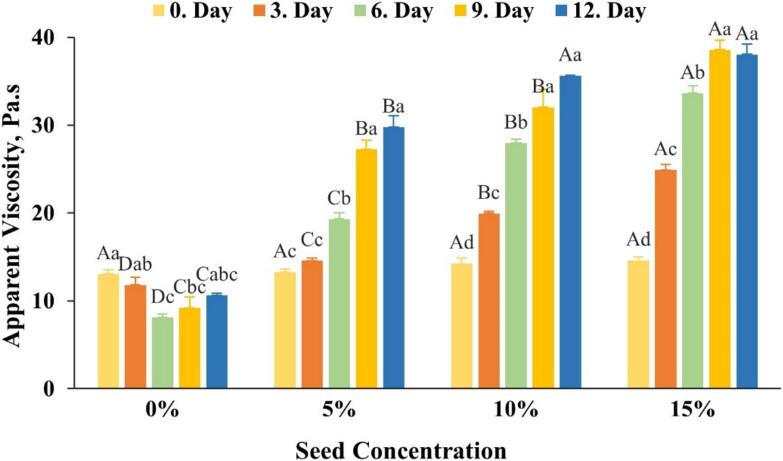
Apparent viscosity (at 50 s^−1^ shear rate) values of the honey samples at different seed concentrations and storage times. Uppercase letters indicate significant differences among seed concentrations, while lowercase letters indicate significant differences among storage days (*p* < 0.05).

The rheological properties of the honey samples changed with the storage time (i.e., 0–12th day) and the concentrations of the seeds (i.e., 0 %–15 %). The fluidity of the honey decreased remarkably as a result of crystallization. Therefore, the parameters related to the flow curve were not given in the study. The apparent viscosity at 50 s^−1^ was considered in the present study, as this shear rate is widely accepted in the literature as a reasonable approximation of the oral shear conditions. Popa Nita et al. (2013) claim that while the precise range of shear rates in the mouth during mastication and swallowing is not definitively known, the shear rate of 50 s^−1^, as advised by the National Dysphagia Diet (NDD), is thought to reflect the in-mouth handling of the bolus with fair accuracy. According to the apparent viscosity results in Fig. 1, there is a significant difference (*p* < 0.05) among the apparent viscosity at 50 s^−1^ values which increased from 13.1 to 38.5 Pa.s (194 % ↑) on increasing the seed concentrations and storage time from 0 to 15 % and 0–12th day, respectively. Likewise, the results for the apparent viscosity values of all other treatment groups at seed concentrations of 5–10 % for 3–9 days of storage also delineated significant (*p* < 0.05) increase in apparent viscosities (Fig. 1). (Conforti et al., 2006) proposed that the initially Newtonian flow behaviour of honey transited into a pseudoplastic/ shear-thinning fluid flow behaviour, which could be attributed to the crystallization process occurring within the honey samples itself. As crystallization progresses, the honey becomes more solidified, leading to increased resistance to flow. Fig. 1 also illustrates that for honey samples supplemented with seeds at concentrations of 5 %, 10 %, and 15 %, the highest apparent viscosity values were attained on the 9th and 12th days, with no significant disparity between these two time points. Consequently, it may be inferred that a storage duration of 9 days is sufficient to achieve the desired level of crystallization in the honey samples. It can also be concluded that the concentration of the seed is vital to achieve the desired apparent viscosities in the final product. Specifically, when aiming for an apparent viscosity value of approximately 40 Pa·s depending on the usage purpose of the crystallized honey, a 10 % seed concentration is more advantageous than a 5 % concentration. An earlier study by Chen et al. (2009) reported that a 0.1 % amount of nuclei in the form of glucose powder is enough for achieving the desired crystallization in honey rather than traditional method as addition of honey seeds at concentrations of 5–10 %. According to this, it can be recommended that increasing the amount of seeds may not be the best for optimizing the crystallization process; however, the differences in the desired crystallization rates could be due to the type or form of sugars like glucose.

**Fig. 2:**
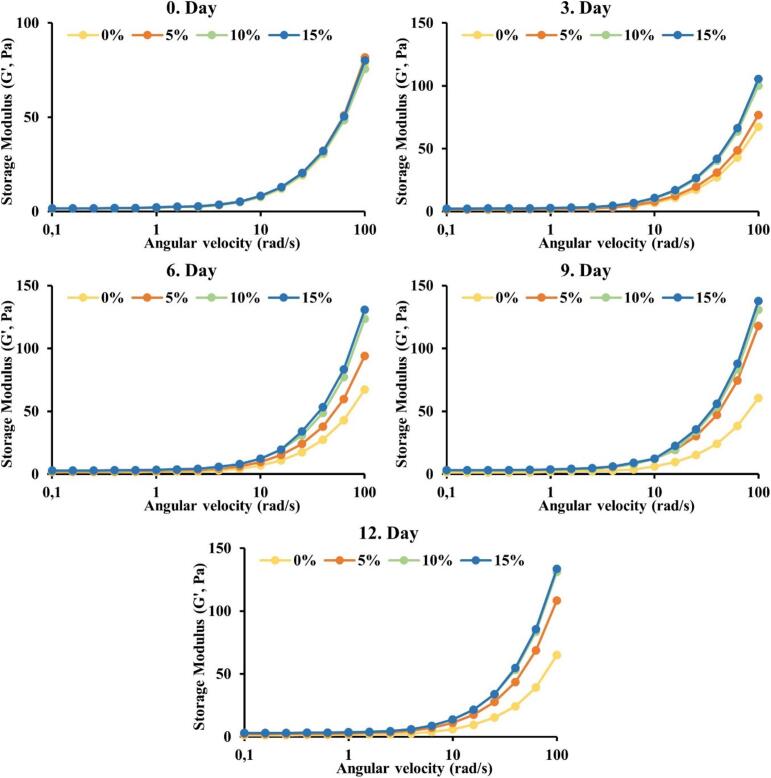
Effect of seed concentration and storage time on the storage modulus (*G'*) of the honey samples.

**Fig. 3:**
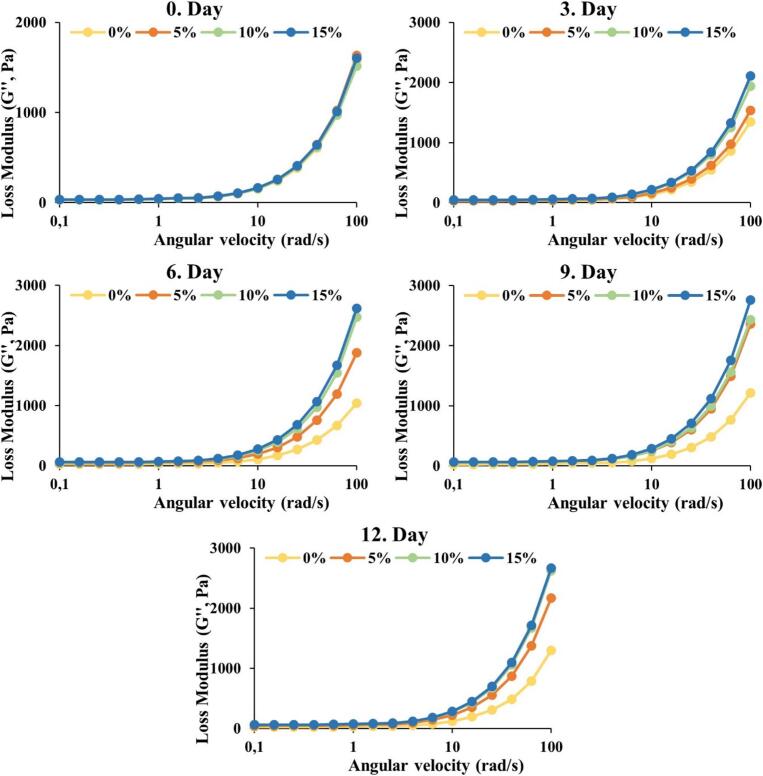
Effect of seed concentration and storage time on the loss modulus (*G"*) of the honey samples.

As it can be seen in Fig. 2, as the angular velocity increases, the storage modulus (*G'*) increases, and these results are parallel to the literature (Ahmed et al., 2007; Dobre et al., 2012). It can fairly be said that at the beginning there is no significant difference between the treatment groups; however, as the time goes, the storage modulus (*G'*) which shows elastic behaviour of the samples, significantly (*p* < 0.05) increased. Also, the samples containing 10–15 % seed honey exhibited a significantly (*p* < 0.05) higher storage modulus compared with the 0–5 % seed honey. However, on the 6th day of storage, the differences between 0 % and 5 % seed concentrations notably increased due to the concentration differences of the seeds added in the honey samples. From this information, it can be thought that the storage modulus is related to the crystallization process of honey and is affected by the seed concentrations. Likewise, up to 9th day of storage, the differences between *G'* values at higher angular velocities of 0 and 5 % seed honey added samples increased. On the 9th and 12th days, slight but non-significant (*p* < 0.05) increases were observed in the *G'* values  of these two groups. The results for the loss modulus of (*G"*) as depicted in Fig. 3, elucidated non-significant differences in *G′′ on the* 0th day of storage. However, the results elucidated that there were significant differences in *G"* values  between control and seed-supplemented samples as of the 3rd day of storage. Peak *G′′ values, as in G′* values, occurred on the 9th day and there was no statistically significant change on the 12th day. The highest *G"* values were recorded as 2756 Pa on the 9th day of storage for the 15 % seed concentration treatment. Comparable findings for *G′′* values were also reported by an earlier study by (Kurt et al., 2020) wherein 10 days of honey storage with seed concentrations of 5–10 % resulted in higher *G′′* values of up to 2000 Pa. The increase in apparent viscosity and the *G'* observed in our study are consistent with the findings of Schiassi et al. (2021), who reported that crystallization directly affects the rheological behaviour of honey. As crystallization progresses, honey becomes more solidified, resulting in increased resistance to flow. This is reflected in the apparent viscosity and storage modulus measurements, where honey samples with higher crystallization levels exhibited greater viscosity and elastic properties.

### 3.2 Textural properties

The firmness values of the honey samples ranged from 21.02 to 24.86 g throughout the 12-day storage period (Table 1). Seed concentration did not cause significant changes in firmness during 0-6th day storage period (*p* > 0.05). On the 9th and 12th days, samples with 5, 10, and 15 % seed concentrations exhibited significantly lower firmness compared with the control (*p* < 0.05). On the other hand, no significant changes (p > 0.05) were observed in firmness values within each sample group during storage. According to (Conforti et al., 2006), high firmness values can be a result of the crystallization process in the honey samples and adhesivity, which is a work for overcoming the forces between materials and food surfaces and is ultimately linked to the firmness. In another study, Suriwong et al. (2021) demonstrated that with extended storage, the degree of crystallization increases, which is correlated with increased firmness.

**Table 1:** Effect of seed concentration and storage time on the textural properties (firmness and work of shear) of honey samples.

Textural Properties		Seed Concentration			
	Days	0 %	5 %	10 %	15 %
Firmness (g)	0	23.82 ± 1.25^Aa^	24.01 ± 0.69^Aa^	23.22 ± 1.40^Aa^	22.92 ± 0.75^Aa^
	3	23.12 ± 2.38^Aa^	22.95 ± 2.29^Aa^	21.55 ± 1.89^Aa^	21.20 ± 1.79^Aa^
	6	23.37 ± 0.57^Aa^	21.70 ± 2.32^Aa^	21.70 ± 1.96^Aa^	21.02 ± 0.44^Aa^
	9	24.86 ± 0.17^Aa^	23.33 ± 0.57^Ba^	23.32 ± 0.68^Ba^	22.43 ± 0.19^Ba^
	12	24.40 ± 0.11^Aa^	21.97 ± 0.41^BCa^	22.43 ± 0.69^Ba^	21.37 ± 0.17^Ca^
Work of Shear (g.s)	0	5.41 ± 0.22^Ab^	5.18 ± 0.37^Ab^	5.42 ± 0.26^Aab^	4.98 ± 0.19^Ab^
	3	5.61 ± 0.19^Ab^	5.32 ± 0.21^Ab^	5.51 ± 0.64^Aab^	5.01 ± 0.34^Ab^
	6	5.90 ± 0.43^Ab^	5.45 ± 0.28^ABb^	5.05 ± 0.28^BCb^	4.79 ± 0.12^Cb^
	9	6.84 ± 0.34^Aa^	6.28 ± 0.08^ABa^	6.16 ± 0.31^BCa^	5.68 ± 0.38^Ca^
	12	6.79 ± 0.37^Aa^	6.18 ± 0.12^ABa^	5.98 ± 0.78^ABab^	5.70 ± 0.24^Ba^

Means are expressed as ± S.D. from triplicate measurements. Uppercase letters indicate the effect of seed concentration, while lowercase letters represent the effect of storage time (in days).

As the storage time increased from 0 to 12 days, the work of shear values also increased for all treatments (p < 0.05) i.e., 5.4 to 6.8, 5.2 to 6.2, 5.4 to 5.9 and 4.9 to 5.7 g.s for 0, 5, 10, and 15 % supplementation levels, respectively (Table 1). Higher seed concentrations (notably 15 %) typically resulted in lower work of shear values compared to the control, especially after 6 days of storage (p < 0.05). In almost all seed concentrations, work of shear values exhibited no significant change until 6th day but increased markedly by 9th day, remaining constant on the 12th day. These findings indicate that both seed concentration and storage time affect the spreadability of honey, with longer storage and higher seed concentrations reducing shear resistance. As per this data, it can be suggested that increasing the seed concentration in honey resultantly decreases the storage time for achieving the desired spreadability for honey samples. It is reasonable to think that more crystal addition in the beginning helps the sugar crystalize faster; therefore, a creamy structure occurs in the early days of storage.

### 3.3 Colour properties

The L* values of the honey samples significantly increased with an increase in both seed concentration and storage time (*p* < 0.05) (Table 2). The *L*^⁎^ values elucidated significant (p < 0.05) increase from 23 to 39, 21 to 38, 25 to 48, 28 to 51 and 32 to 55, respectively, on increasing the concentrations of seed addition from 0 %–15 %. Similarly, a positive correlation was also observed on the *L*^⁎^ values of honey samples wherein the *L*^⁎^ values changed between 23 and 32, 28 and 37, 37 and 51, and 39 and 55, respectively for 0, 5, 10 and 15 % seed concentrations at 0–12 days. Similar trends were observed by Schiassi et al. (2021) and Dettori et al. (2018), where the L* value, representing lightness, increased over storage time. An increase in the *L*^⁎^ values of the honey samples can be an indicator of crystallization in the samples because the natural colour of the honey can be affected by the storage time and conditions due to changes like crystallization and de-crystallization (Önür et al., 2018; Piotraszewska-Pająk & Gliszczyńska-Świgło, 2015). Glucose crystals are pure and white in nature (Hamdan, 2010), so the increase in lightness of the honey samples on the addition of seed concentrations and storage could be claimed as an indicator of crystallization.

**Table 2:** Effect of seed concentration and storage time on the *L*^⁎^ values of honey samples.

Days	0 %	5 %	10 %	15 %
0	23.32 ± 0.13^Dc^	28.73 ± 1.19^Cb^	36.74 ± 3.30^Bd^	39.27 ± 0.30^Ad^
3	20.74 ± 0.92^Dd^	26.93 ± 0.52^Cbc^	34.61 ± 0.09^Bd^	38.21 ± 0.59^Ae^
6	24.48 ± 1.68^Cc^	24.77 ± 1.06^Cc^	45.17 ± 0.35^Bc^	48.26 ± 0.48^Ac^
9	28.24 ± 1.93^Db^	35.08 ± 3.15^Ca^	47.78 ± 0.81^Bb^	51.30 ± 0.29^Ab^
12	32.38 ± 0.70^Da^	37.60 ± 1.00^Ca^	50.69 ± 0.81^Ba^	55.12 ± 0.45^Aa^

Means are expressed as ± S.D. from triplicate measurements. Uppercase letters indicate the effect of seed concentration, while lowercase letters represent the effect of storage time (in days).

### 3.4 Microscopic images

**Fig. 4:**
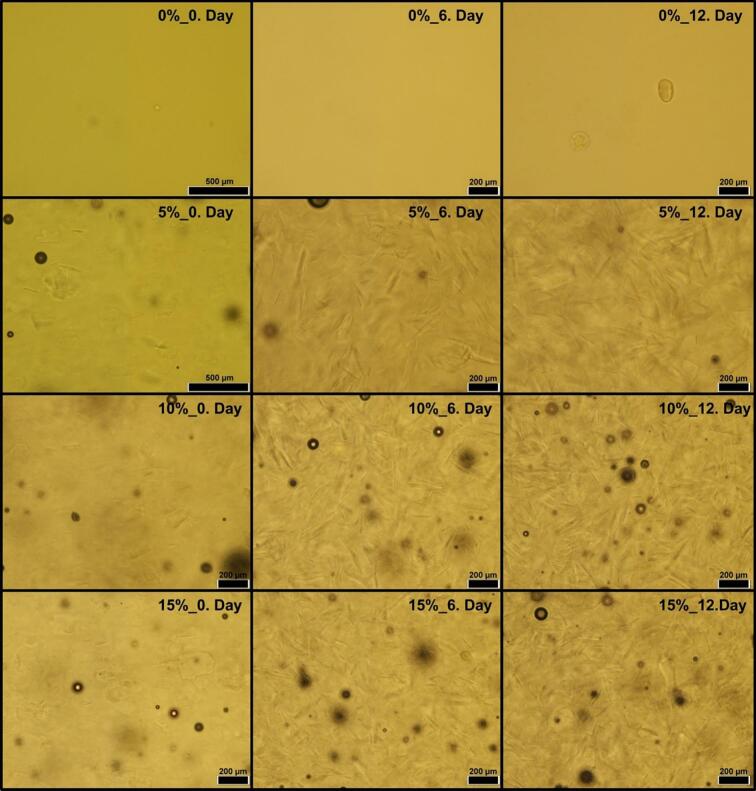
Microscopic images of the honey samples at different seed concentration and storage time.

As per the results for the microscopic examination of the crystals, it can be observed in Fig. 4, there is no crystallization in all samples of the control (i.e., without seeds) even on the 12th day of storage. However, on the addition of seeds concentrations, the crystal formation in all honey samples increased (Fig. 4). On the 6th and 12th days of storage, it was recorded that 10 % and 15 % addition of seeds in all honey samples increased the size and magnitudes of crystals higher than the 5 % seed addition. However, among the treatments, 10 % and 15 % showed non-significant (*p* < 0.05) differences in the results for the microscopic crystals. In our study, microscopic analysis revealed the formation and development of crystals as storage time progressed, similar to findings reported by Conforti et al. (2006) and Schiassi et al. (2021). Earlier studies have also exhibited comparable needle and flat-plate-shaped honey crystals as revealed in the microscopic examination of the present study. According to the available literature, honey crystals can be of needle shaped, flat plate or star shaped depending upon the growth stage, nucleation, temperature and storage duration (Bhandari et al., 1999).

## 4 Conclusion

This study demonstrates that seed concentration and storage time significantly influence the rheological, textural, and crystallization properties of creamed honey. Optimal quality was observed at 10 % seed concentration and 9 days of storage, producing a desirable spreadable consistency with enhanced viscosity and crystal formation. These findings support the potential for targeted process optimization in commercial creamed honey production. Future research should explore the effects of seed blends and processing variables, such as temperature, to refine product quality. The adaptability of creamed honey's texture and flow behaviour also presents valuable opportunities for its use in gastronomy, including as a filling or coating agent in diverse food applications.

## CRediT authorship contribution statement

**Duygu Ozmen:** Writing – review & editing, Writing – original draft, Methodology, Formal analysis, Conceptualization. **Hüseyin Demircan:** Writing – review & editing, Writing – original draft, Methodology, Formal analysis. **Rusen Metin Yildirim:** Writing – review & editing, Writing – original draft, Methodology, Formal analysis, Conceptualization. **Muhammad Waseem:** Writing – review & editing, Writing – original draft, Methodology, Formal analysis. **Hakan Basdogan:** Writing – review & editing, Writing – original draft, Methodology, Formal analysis. **Omer Said Toker:** Writing – review & editing, Writing – original draft, Supervision, Investigation, Conceptualization. **Crossby Osei Tutu:** Writing – review & editing, Visualization, Validation, Software, Resources, Methodology, Investigation, Formal analysis.

## Declaration of competing interest

The authors declare that they have no known competing financial interests or personal relationships that could have appeared to influence the work reported in this paper.

## Data Availability

The data associated with this study are included in article/referenced in article.
